# Lumped Parameter Thermal Network Modeling and Thermal Optimization Design of an Aerial Camera

**DOI:** 10.3390/s24123982

**Published:** 2024-06-19

**Authors:** Yue Fan, Wei Feng, Zhenxing Ren, Bingqi Liu, Dazhi Wang

**Affiliations:** 1 College of Mechanical Engineering, Chengdu University, Chengdu 610106, China; fengwei@cdu.edu.cn (W.F.); renzhenxing@cdu.edu.cn (Z.R.); liubingqi@cdu.edu.cn (B.L.); 2 Institute of Optics and Electronics, Chinese Academy of Sciences, Chengdu 610209, China; wangdazhi@ioe.ac.cn

**Keywords:** aerial camera, remote sensor, lumped parameter thermal network, thermal resistance, heat leakage, thermal design

## Abstract

The quality of aerial remote sensing imaging is heavily impacted by the thermal distortions in optical cameras caused by temperature fluctuations. This paper introduces a lumped parameter thermal network (LPTN) model for the optical system of aerial cameras, aiming to serve as a guideline for their thermal design. By optimizing the thermal resistances associated with convection and radiation while considering the camera’s unique internal architecture, this model endeavors to improve the accuracy of temperature predictions. Additionally, the proposed LPTN framework enables the establishment of a heat leakage network, which offers a detailed examination of heat leakage paths and rates. This analysis offers valuable insights into the thermal performance of the camera, thereby guiding the refinement of heating zones and the development of effective active control strategies. Operating at a total power consumption of 26 W, the thermal system adheres to the low-power limit. Experimental data from thermal tests indicate that the temperatures within the optical system are maintained consistently between 19 °C and 22 °C throughout the flight, with temperature gradients remaining below 3 °C, satisfying the temperature requirements. The proposed LPTN model exhibits swiftness and efficacy in determining thermal characteristics, significantly facilitating the thermal design process and ensuring optimal power allocation for aerial cameras.

## 1. Introduction

Aerial cameras, serving as crucial remote sensors, are extensively utilized in both military and civilian domains for their ability to provide all-weather, real-time, and high-resolution images of ground targets [1,2,3,4]. In the challenging and ever-changing environment in which aerial cameras operate, temperature disturbance is a major factor that significantly impacts imaging quality. These disturbances can cause uneven temperature distributions within the optical system, leading to the thermal deformation of optical components and assembly structures. These thermal distortions can result in various imaging artifacts such as hot/cold spots, blurring, and thermal drift, ultimately affecting the overall image quality of aerial cameras.

To ensure high-resolution imaging, a precise thermal control optimization design is paramount for aerial cameras. The primary objective is to maintain the temperature of the optical system within a specified range across various flight conditions [5,6,7,8]. Given the limited energy resources on aircrafts, the thermal control system must prioritize energy efficiency, aiming to minimize consumption without compromising system reliability. Consequently, a comprehensive understanding of the camera’s thermal characteristics is imperative, enabling the rational allocation of thermal control resources and enhancing control accuracy [9,10,11].

The complex internal structure of aerial imaging systems, coupled with the changing external thermal environment during flight and the need to arrange instruments according to actual flight mission requirements, make the thermal analysis and temperature calculation of their optical and structural components quite challenging. Analytical solutions for these problems can be difficult to come by, as they require a deep understanding of the underlying physics and mathematical models involved in thermal control optimization design. In many cases, numerical approximation methods are used to solve these problems.

The finite element method (FEM) was originally developed for structural mechanics and gradually expanded to fields such as fluid mechanics and heat transfer in the 1970s [12,13]. In recent years, the FEM has gained widespread use in the thermal structural analysis of ground-based, aerial, and space-based optical imaging equipment, which has led to significant improvements in the accuracy and efficiency of thermal control and structural optimization designs. Ravinder et al. [14] used the finite element structural model to predict the temperature-induced mechanical deformations of a mirror blank for a solar telescope. Liu et al. [15] established an FE thermal model for an aerial camera to calculate the model’s temperature distribution according to the boundary conditions. Gao et al. [16] verified the rationality of a thermal control scheme for an infrared aerial camera by a finite element thermal analysis. Borden et al. [17] built thermal and structural FE models of a balloon-based imaging system to simulate system performance and ultimately inform the final pointing stability predictions. Cui et al. [18] introduced a thermal model updating method using the Kriging model as a surrogate model to optimize the thermal design parameters of a solar spectrometer, rather than directly iterating a finite element analysis.

The lumped parameter thermal network (LPTN) method is another reliable approach that was initially commonly used for temperature estimations in spacecrafts. Ishimoto et al. [19] verified the LPTN math models of a spacecraft thermal control system using ground-based tests based on the Kalman filtering method. Weng et al. [20] established the thermal network model of a spacecraft and corrected partial coefficients by simplifying the radiation relationships of a mathematical model based on Least Square Estimation. Liu et al. [21] proposed the Variables–Parameters Coefficient method and Monte Carlo Ray Tracing method to correct the thermal network model of a spacecraft.

Characterized by its intuitive physical concepts, the LPTN method has been utilized for the thermal analysis of ground- and air-based optical systems. Edeson et al. [22] established a thermal network model specified by conductive, radiative, and convective couplings between nodes for an infrared camera using the software ESATAN, and steady-state temperature distributions were obtained as temperature loads of the structural analysis. Xue et al. [23] developed a thermal network model for an aerial camera to estimate its thermal behavior before an integrated analysis and to provide a reliable initial design reference for the thermal control system. In our previous work [24], we developed a general heat leakage network model for the lenses and optical window of an aerial camera to estimate its maximum thermal leakages under extreme operating conditions and designed heating loops and heating power according to the calculated results. However, the model’s precision was insufficient, and the study remained at the simulation stage with no thermal control experiments conducted.

Previous thermal network models of aerial cameras fell short in several key areas, most notably in the lack of the optimization of convection and radiation thermal resistances tailored to the specific characteristics of the camera. Moreover, given the finite resources available in aerial applications, previous studies lacked an effective strategy for optimizing the allocation of thermal control energy.

The enhanced lumped parameter thermal network (LPTN) model presented in this study addresses these gaps by introducing a refined approach that accounts for the unique internal structure of the aerial camera. By optimizing the thermal resistances related to convection and radiation, this model aims to improve the precision of thermal analysis, ultimately leading to more accurate predictions of the camera’s thermal behavior.

Furthermore, the heat leakage network established within the LPTN framework provides a comprehensive understanding of the heat leakage paths and rates within the optical system. This analysis offers profound insights into the camera’s thermal performance, enabling targeted thermal optimization measures to be implemented. By leveraging the heat leakage analysis results, the design of active thermal control is optimized to manage heat dissipation and maintain the camera’s temperature within the desired range. This not only ensures image resolution but also reduces energy expenditures, contributing to the sustainability of long-duration missions.

## 2. Introduction of the Aerial Camera

### 2.1. Thermal Environment

**Figure 1:**
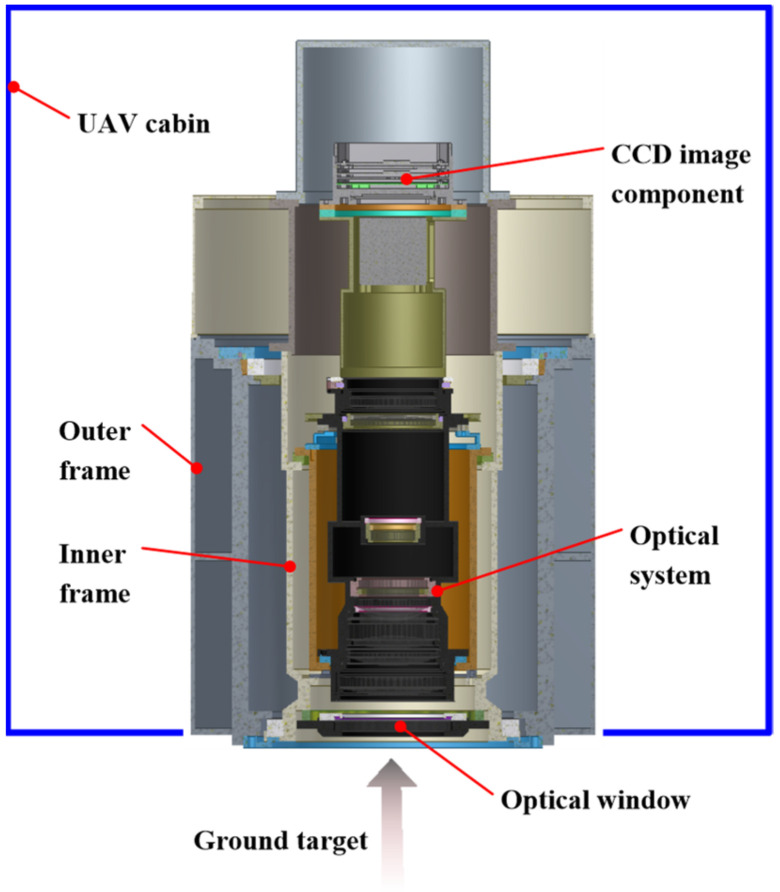
The load configuration of the aerial camera.

The aerial camera is installed in an unmanned aerial vehicle (UAV), as illustrated in Figure 1. The optical window points vertically towards the ground target, and its outer surface is in contact with the external environment directly. The flight attitude of the UAV is 9000 m, and the ambient temperature is −38.5 °C when the ground temperature is 20 °C. The total power that is allocated to the thermal control system of the camera is 100 W.

### 2.2. Effects of Temperature Variations on Optical Systems

The variations in temperature within optical systems include both fluctuations in temperature levels and temperature gradients. Specifically, alterations in temperature levels can trigger changes in crucial parameters such as the refractive index of optical materials, the refractive index of air, the curvature radius and center thickness of lenses, and the lens center distance. These changes culminate in modifications to the focal length of the system. Meanwhile, temperature gradients within the lens can lead to issues like defocusing, spherical aberration, coma, and astigmatism in the optical system.

Through a comprehensive integrated thermal/structural/optical analysis, the modulation transfer function (MTF) variations at a spatial frequency of 70 lp/mm under varying temperature levels and temperature gradients have been derived and are presented in Table 1. It is evident that, following focusing, the temperature level can meet the imaging quality requirements within the range of −20 °C to 20 °C. Since focusing is only performed on the ground, the development of thermal control indicators is based on ground temperatures. Specifically, when the ground temperature is 20 °C, it is advisable that the ideal operating temperature for obtaining high-quality images with the optical system falls within a range of (20 ± 5) °C while maintaining radial and axial temperature gradients below 5 °C.

### 2.3. Experimental Aerial Camera

**Figure 2:**
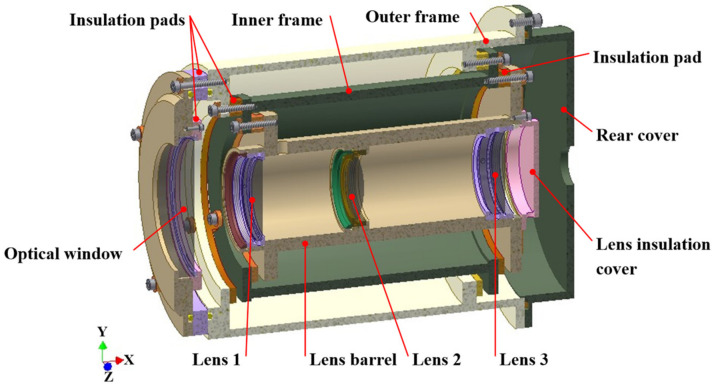
Structural framework of the experimental aerial camera.

Based on the framework structure of the original prototype of the aerial camera and combining this with the preliminary design for thermal control, an experimental aerial camera is proposed, as shown in Figure 2. The experimental aerial camera (hereinafter referred to as the aerial camera) features rotational symmetry along the *X*-axis, with an outer dimension of Φ173 mm × 258 mm, including an optical window, three sets of lenses, a lens barrel, inner frame, outer frame, and rear cover. The lens insulation cover is set to simulate the thermal flow impact of the CCD image component on the lens assembly.

The outer surface of the optical window, which points towards the ground, loses a considerable amount of heat to the external environment, causing a rapid decline in temperature and an axial temperature difference, which has an impact on the temperature of Lens 1. Additionally, the lens assembly and framework structure experience significant heat transfer through conduction, convection, and radiation, resulting in a rapid decrease in their temperature level and large radial and axial temperature differences. Therefore, thermal control for the optical system aims to improve the temperature level and reduce the temperature difference. A range of passive thermal control measures are implemented, including installing insulation pads between frame structures and coating an ITO film on the outer surface of the optical window.

## 3. LPTN Modeling

### 3.1. Thermal Network Node Model

The LPTN method relies on the analogy between heat transfer processes and electrical conduction processes. First of all, the primary components of the physical model are partitioned into temperature units that exhibit uniform temperatures and heat fluxes. The thermal properties of each unit are concentrated on its centroid, designated as the node. Each node is characterized by lumped thermal properties: the temperature T and heat capacity C. The positioning and the number of nodes in the thermal network model have a significant impact on the accuracy and computational time of the results. The overall objective is to minimize the number of nodes while still accurately capturing the primary thermal characteristics of the aerial camera.

**Figure 3:**
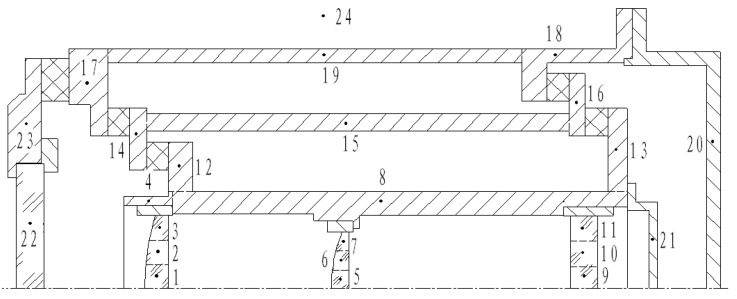
Thermal network node model of the aerial camera.

To simplify the model, structures such as screws, holes, and threads that have little effect on the temperature are ignored. Components with high thermal conductivity are sparsely divided into nodes, such as the lens barrel, inner frame, and outer frame. For the lens with a small thickness, the temperature difference in the radial direction is significantly larger than that in the axial direction, thus the nodes of the lens should be densely divided along the radial direction. As the outer surface of the optical window is uniformly influenced by external heat flows, resulting in small temperature differences in both the radial and axial directions, the optical window can be treated as a single node. Therefore, a thermal network model with 23 nodes is established for the aerial camera, as shown in Figure 3. The 24th node represents the external environment. The locations and physical parameters of the nodes are illustrated in Table 2.

### 3.2. Thermal Network Mathematical Model

**Figure 4:**
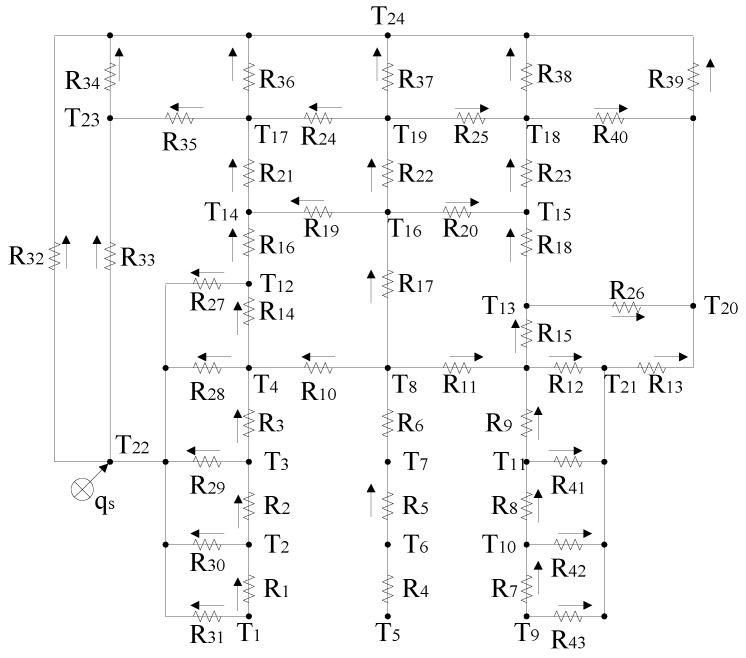
Thermal network of the aerial camera.

The thermal network of the aerial camera is constructed by connecting nodes using thermal resistances, as depicted in Figure 4. The arrows indicate the direction of heat flow, and *q_s_* represents the external heat flow received by the outer surface of the optical window.

Based on the similarity between heat transfer and conduction processes, and utilizing insights from Kirchhoff’s current law and Ohm’s law, the thermal balance equations for each node can be written as follows:

(1) $${\left(mc\right)}_{1}\frac{dT_{1}}{d\tau }=-\frac{T_{1}-T_{2}}{R_{1}}-\frac{T_{1}-T_{22}}{R_{31}}$$

(2) $${\left(mc\right)}_{2}\frac{dT_{2}}{d\tau }=\frac{T_{1}-T_{2}}{R_{1}}-\frac{T_{2}-T_{3}}{R_{2}}-\frac{T_{2}-T_{22}}{R_{30}}$$

(3) $${\left(mc\right)}_{3}\frac{dT_{3}}{d\tau }=\frac{T_{2}-T_{3}}{R_{2}}-\frac{T_{3}-T_{4}}{R_{3}}-\frac{T_{3}-T_{22}}{R_{29}}$$

(4) $${\left(mc\right)}_{4}\frac{dT_{4}}{d\tau }=\frac{T_{3}-T_{4}}{R_{3}}+\frac{T_{8}-T_{4}}{R_{10}}-\frac{T_{4}-T_{12}}{R_{14}}-\frac{T_{4}-T_{22}}{R_{28}}$$

(5) $${\left(mc\right)}_{5}\frac{dT_{5}}{d\tau }=-\frac{T_{5}-T_{6}}{R_{4}}$$

(6) $${\left(mc\right)}_{6}\frac{dT_{6}}{d\tau }=\frac{T_{5}-T_{6}}{R_{4}}-\frac{T_{6}-T_{7}}{R_{5}}$$

(7) $${\left(mc\right)}_{7}\frac{dT_{7}}{d\tau }=\frac{T_{6}-T_{7}}{R_{5}}-\frac{T_{7}-T_{8}}{R_{6}}$$

(8) $${\left(mc\right)}_{8}\frac{dT_{7}}{d\tau }=\frac{T_{7}-T_{8}}{R_{6}}+\frac{T_{11}-T_{8}}{R_{11}+R_{9}}-\frac{T_{8}-T_{4}}{R_{10}}-\frac{T_{8}-T_{13}}{R_{11}+R_{15}}-\frac{T_{8}-T_{16}}{R_{17}}-\frac{T_{8}-T_{21}}{R_{11}+R_{12}}$$

(9) $${\left(mc\right)}_{9}\frac{dT_{9}}{d\tau }=-\frac{T_{9}-T_{10}}{R_{7}}-\frac{T_{9}-T_{21}}{R_{43}}$$

(10) $${\left(mc\right)}_{10}\frac{dT_{10}}{d\tau }=\frac{T_{9}-T_{10}}{R_{7}}-\frac{T_{10}-T_{11}}{R_{8}}-\frac{T_{10}-T_{21}}{R_{42}}$$

(11) $${\left(mc\right)}_{11}\frac{dT_{11}}{d\tau }=\frac{T_{10}-T_{11}}{R_{8}}-\frac{T_{11}-T_{8}}{R_{9}+R_{11}}-\frac{T_{11}-T_{13}}{R_{9}+R_{15}}-\frac{T_{11}-T_{21}}{R_{9}+R_{12}}-\frac{T_{11}-T_{21}}{R_{41}}$$

(12) $${\left(mc\right)}_{12}\frac{dT_{12}}{d\tau }=\frac{T_{4}-T_{12}}{R_{14}}-\frac{T_{12}-T_{14}}{R_{16}}-\frac{T_{12}-T_{22}}{R_{27}}$$

(13) $${\left(mc\right)}_{13}\frac{dT_{13}}{d\tau }=\frac{T_{8}-T_{13}}{R_{11}+R_{15}}+\frac{T_{11}-T_{13}}{R_{9}+R_{15}}+\frac{T_{21}-T_{13}}{R_{12}+R_{15}}-\frac{T_{13}-T_{15}}{R_{18}}-\frac{T_{13}-T_{20}}{R_{26}}$$

(14) $${\left(mc\right)}_{14}\frac{dT_{14}}{d\tau }=\frac{T_{12}-T_{14}}{R_{16}}+\frac{T_{16}-T_{14}}{R_{19}}-\frac{T_{14}-T_{17}}{R_{21}}$$

(15) $${\left(mc\right)}_{15}\frac{dT_{15}}{d\tau }=\frac{T_{13}-T_{15}}{R_{18}}+\frac{T_{16}-T_{15}}{R_{20}}-\frac{T_{15}-T_{18}}{R_{23}}$$

(16) $${\left(mc\right)}_{16}\frac{dT_{16}}{d\tau }=\frac{T_{8}-T_{16}}{R_{17}}-\frac{T_{16}-T_{14}}{R_{19}}-\frac{T_{16}-T_{15}}{R_{20}}-\frac{T_{16}-T_{19}}{R_{22}}$$

(17) $${\left(mc\right)}_{17}\frac{dT_{17}}{d\tau }=\frac{T_{14}-T_{17}}{R_{21}}+\frac{T_{19}-T_{17}}{R_{24}}-\frac{T_{17}-T_{23}}{R_{35}}-\frac{T_{17}-T_{24}}{R_{36}}$$

(18) $${\left(mc\right)}_{18}\frac{dT_{18}}{d\tau }=\frac{T_{15}-T_{18}}{R_{23}}+\frac{T_{19}-T_{18}}{R_{25}}-\frac{T_{18}-T_{20}}{R_{40}}-\frac{T_{18}-T_{24}}{R_{38}}$$

(19) $${\left(mc\right)}_{19}\frac{dT_{19}}{d\tau }=\frac{T_{16}-T_{19}}{R_{22}}-\frac{T_{19}-T_{17}}{R_{24}}-\frac{T_{19}-T_{18}}{R_{25}}-\frac{T_{19}-T_{24}}{R_{37}}$$

(20) $${\left(mc\right)}_{20}\frac{dT_{20}}{d\tau }=\frac{T_{13}-T_{20}}{R_{26}}+\frac{T_{21}-T_{20}}{R_{13}}+\frac{T_{18}-T_{20}}{R_{40}}-\frac{T_{20}-T_{24}}{R_{39}}$$

(21) $${\left(mc\right)}_{21}\frac{dT_{21}}{d\tau }=\frac{T_{8}-T_{21}}{R_{11}+R_{12}}+\frac{T_{11}-T_{21}}{R_{9}+R_{12}}+\frac{T_{11}-T_{21}}{R_{41}}-\frac{T_{21}-T_{13}}{R_{12}+R_{15}}-\frac{T_{21}-T_{20}}{R_{13}}$$

(22) $$\begin{array}{l} {\left(mc\right)}_{22}\frac{dT_{22}}{d\tau }=q_{s}+\frac{T_{1}-T_{22}}{R_{31}}+\frac{T_{2}-T_{22}}{R_{30}}+\frac{T_{3}-T_{22}}{R_{29}}+\\ \;\;\;\;\;\;\;\frac{T_{4}-T_{22}}{R_{28}}+\frac{T_{12}-T_{22}}{R_{27}}-\frac{T_{22}-T_{23}}{R_{33}}-\frac{T_{22}-T_{24}}{R_{32}} \end{array}$$

(23) $${\left(mc\right)}_{23}\frac{dT_{23}}{d\tau }=\frac{T_{17}-T_{23}}{R_{35}}+\frac{T_{22}-T_{23}}{R_{33}}-\frac{T_{23}-T_{24}}{R_{34}}$$

where (*mc*)*_i_* (*i* = 1~23) is the thermal capacity, *T*_1_~*T*_24_ are the node temperatures, and *R*_1_~*R*_43_ are the heat transfer resistances between nodes.

There are four types of thermal resistance, namely *R*_cd_, *R*_ct_, *R_c_*_v_, and *R*_rad_, which correspond to the conductive, contact, convective, and radiation thermal resistance, respectively. The *R*_cd_ and *R*_ct_ can be calculated according to thermal resistance principles [25].

A. Convective thermal resistance *R*_cv_

In the internal structure of aerial cameras, the convective heat transfer between surfaces can be simplified into two cavity convection types: a rectangular cavity or concentric cylinder.

**Figure 5:**
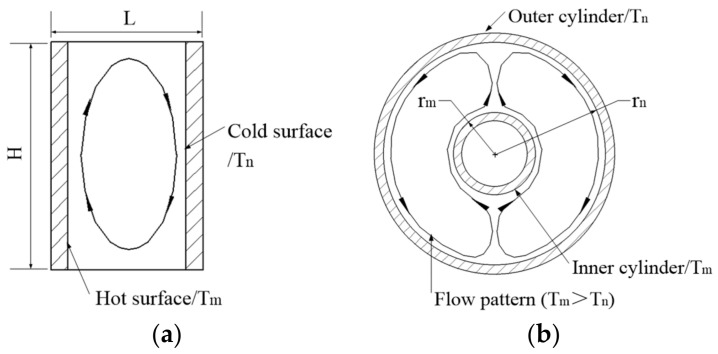
The flow pattern of convective heat transfer: (**a**) rectangular cavity; (**b**) concentric cylinder.

Figure 5a illustrates the flow pattern from the hot surface to the cold surface in a rectangular cavity. The convective heat transfer coefficient is calculated using the following equation [25]:

(24) $$h=0.22{\left(\frac{\mathrm{Pr}}{0.2+\mathrm{Pr}}Ra_{L}\right)}^{0.28}{\left(\frac{H}{L}\right)}^{-1/4}\frac{\lambda_{f}}{L}$$

where Pr is the Prandtl number, *Ra_L_* is the Rayleigh number, and *H* and *L* are the height and width of the rectangular cavity, respectively.

By refining *Ra_L_* and the fluid volume expansion coefficient, Equation (24) can be rewritten as

(25) $$h=0.267{\left(\frac{\mathrm{Pr}gL^{3}}{\alpha \nu (0.2+\mathrm{Pr})}\right)}^{0.28}{\left(\frac{H}{L}\right)}^{-1/4}\frac{\lambda_{f}}{L}{\left|\frac{T_{m}-T_{n}}{T_{m}+T_{n}}\right|}^{0.28}$$

where *g* is gravitational acceleration, *α* is the thermal diffusion coefficient, *ν* is the kinematic viscosity, and *T_m_* and *T_n_* are temperature of the hot surface and the cold surface, respectively.

The flow pattern in a concentric cylinder is shown in Figure 5b, and the heat transfer rate can be expressed as

(26) $$q=\frac{2\pi L\lambda_{eff}(T_{m}-T_{n})}{\ln(r_{n}/r_{m})}$$

where *r_m_* and *r_n_* are the radius of the inner and outer cylinder, respectively, and *λ_eff_* is the effective thermal conductivity of the fluid, which has the following relationship to thermal conductivity *λ_f_*:

(27) $$\lambda_{eff}=\lambda_{f}\cdot 0.386{\left(\frac{\mathrm{Pr}}{0.861+\mathrm{Pr}}\right)}^{1/4}{Ra}_{c}^{1/4}$$

where

(28) $$Ra_{c}=\frac{8g\beta (T_{m}-T_{n}){\left[\ln(r_{n}/r_{m})\right]}^{4}}{\alpha \nu {\left(r_{m}^{-3/5}+r_{n}^{-3/5}\right)}^{5}}$$

where *β* is the fluid volume expansion coefficient.

The convective heat transfer thermal resistance in a concentric cylinder can be derived from Equations (26)–(28) as follows:

(29) $$R_{\mathrm{cv}}=\frac{1}{1.472\pi L\lambda_{f}}{\left[\frac{g\mathrm{Pr}}{\alpha \nu {\left(r_{m}^{-3/5}+r_{n}^{-3/5}\right)}^{5}(0.861+\mathrm{Pr})}\right]}^{-1/4}{\left|\frac{T_{m}+T_{n}}{T_{m}-T_{n}}\right|}^{0.25}$$

B. Radiation thermal resistance *R*_rad_

The radiation heat transfer within aerial cameras can be classified into two main categories: that between concentric cylindrical surfaces and that between coaxial parallel discs.

The radiation thermal resistance between concentric cylindrical surfaces can be written as

(30) $$R_{\mathrm{rad}}=\frac{\frac{1}{\varepsilon_{i}}+\frac{1-\varepsilon_{o}}{\varepsilon_{o}}\left(\frac{r_{i}}{r_{o}}\right)}{\sigma A_{i}(T_{i}^{2}+T_{o}^{2})(T_{i}+T_{o})}$$

where *ε_i_*, *ε_o_*, *r_i_*, *r_o_, T_i_*, and *T*_o_ are the emissivity, radius, and temperature of the inner and outer surfaces, respectively, *σ* is the Stefan–Boltzmann constant, and *A_i_* is the area of the inner surface.

The radiation thermal resistance between coaxial parallel discs can be expressed as

(31) $$R_{\mathrm{rad}}=\frac{\frac{1}{\varepsilon_{i}}+\frac{1}{\varepsilon_{j}}-1}{F_{ij}\sigma A_{i}(T_{i}^{2}+T_{o}^{2})(T_{i}+T_{o})}$$

where *F_ij_* is the radiation angle coefficient, which can be calculated as follows:

(32) $$F_{ij}=\frac{1}{2}\left\{1+\frac{d+r_{j}^{2}}{r_{i}^{2}}-{\left[{\left(1+\frac{d+r_{j}^{2}}{r_{i}^{2}}\right)}^{2}-4{\left(r_{j}/r_{i}\right)}^{2}\right]}^{1/2}\right\}$$

where *r_i_* and *r_j_* are the radius of the two coaxial parallel discs, respectively, and *d* is their axial distance.

The computed values for thermal resistance, ranging from *R*1 to *R*43, are presented in Table A1, which is located in Appendix A.

Assuming a ground temperature of 20 °C, as the UAV elevates to its working altitude of 9000 m at a consistent speed within 2000 s, the temperature change at node 24 can be described as follows:

(33) $$T_{24}=\left\{\begin{array}{ll} 20-0.0293t & \left(0\leq t\leq 2000s\right)\\ -38.5 & \left(t>2000s\right) \end{array}\right.$$

**Figure 6:**
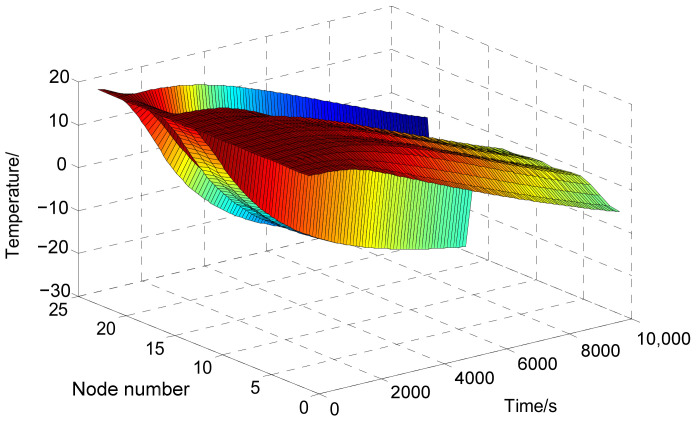
Temperature variations of thermal network nodes.

By substituting the heat capacity values in Table 2 and the thermal resistance values of *R*1 to *R*43 into Equations (1)–(23), a nonlinear differential equation system for the temperature of 23 nodes is obtained. With the initial temperatures of all nodes set to 20 °C, the temperature variations of each node over a period of 10,000 s, spanning from take-off to the photo-shooting stage, are precisely determined by incorporating the boundary conditions outlined in Equation (33) into the nonlinear differential equation system, as illustrated in Figure 6. In the figure, various colors denote different temperatures. As the hues progressively shift from warm to cool tones, the temperature decreases accordingly. As the figure reveals, despite the lens temperature remaining within the (20 ± 5) °C range during the initial phase of camera operation, it gradually exceeds these temperature specifications as the shooting duration increases. Notably, the optical window temperature decreases more rapidly, falling short of meeting the temperature requirements from the very beginning of camera operation. Therefore, it is crucial to implement active thermal controls for both the lens and the optical window to ensure they remain within the desired temperature range.

### 3.3. Heat Leakage Analysis

**Figure 7:**
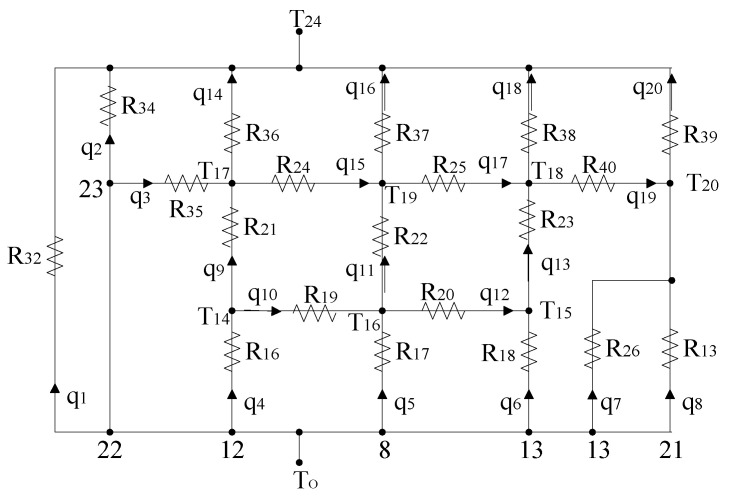
Heat leakage network of the optical system.

To determine effective active thermal control measures and the minimum heating power required to maintain the temperature requirements of the optical system, a heat leakage network for the optical system is established, as illustrated in Figure 7. There are eight heat leakage paths in the optical system; *q*_1_ and *q*_2_ + *q*_3_ are the heat leakage rates of the optical window and window barrel, respectively, and *q*_4_, *q*_5_, *q*_6_ + *q*_7_, and *q*_8_ are the heat leakage rates of the lens barrel.

Based on the established heat leakage network, steady-state thermal equilibrium equations are formulated as follows:

(34) $$T_{o}-T_{24}=\frac{q_{1}}{0.025+1.14\times 10^{-10}(T_{o}^{2}+T_{24}^{2})(T_{o}+T_{24})}$$

(35) $$T_{o}-T_{24}=\frac{q_{2}}{0.11+9.62\times 10^{-10}(T_{o}^{2}+T_{24}^{2})(T_{o}+T_{24})}$$

(36) $$T_{o}-T_{17}=5.12q_{3}$$

(37) $$T_{o}-T_{14}=51.4q_{4}$$

(38) $$T_{o}-T_{16}=\frac{q_{5}}{0.24{\left|\frac{T_{o}-T_{16}}{T_{o}+T_{16}}\right|}^{0.25}+2\times 10^{-10}(T_{o}^{2}+T_{16}^{2})(T_{o}+T_{16})}$$

(39) $$T_{o}-T_{15}=51.3q_{6}$$

(40) $$T_{o}-T_{20}=\frac{q_{7}}{0.07{\left|\frac{T_{o}-T_{20}}{T_{o}+T_{20}}\right|}^{0.28}+3.32\times 10^{-11}(T_{o}^{2}+T_{21}^{2})(T_{o}+T_{21})}$$

(41) $$T_{o}-T_{21}=\frac{q_{6}}{0.02{\left|\frac{T_{o}-T_{21}}{T_{o}+T_{21}}\right|}^{0.28}+1.12\times 10^{-11}(T_{21}^{2}+T_{o}^{2})(T_{21}+T_{o})}$$

(42) $$T_{14}-T_{17}=51.3q_{9}$$

(43) $$T_{14}-T_{16}=0.22q_{10}$$

(44) $$T_{16}-T_{19}=\frac{q_{11}}{0.34{\left|\frac{T_{16}-T_{19}}{T_{16}+T_{19}}\right|}^{0.25}+1.4\times 10^{-10}(T_{16}^{2}+T_{19}^{2})(T_{16}+T_{19})}$$

(45) $$T_{16}-T_{15}=0.22q_{12}$$

(46) $$T_{15}-T_{18}=51q_{13}$$

(47) $$T_{17}-T_{24}=\frac{q_{14}}{0.038+3.02\times 10^{-11}(T_{17}^{2}+T_{24}^{2})(T_{17}+T_{24})}$$

(48) $$T_{17}-T_{19}=0.19q_{15}$$

(49) $$T_{19}-T_{24}=\frac{q_{16}}{0.41+3.24\times 10^{-10}(T_{19}^{2}+T_{24}^{2})(T_{19}+T_{24})}$$

(50) $$T_{19}-T_{18}=0.23q_{17}$$

(51) $$T_{18}-T_{24}=\frac{q_{18}}{0.13+1.07\times 10^{-10}(T_{18}^{2}+T_{24}^{2})(T_{18}+T_{24})}$$

(52) $$T_{18}-T_{20}=0.27q_{19}$$

(53) $$T_{20}-T_{24}=\frac{q_{20}}{0.23+1.82\times 10^{-10}(T_{20}^{2}+T_{24}^{2})(T_{20}+T_{24})}$$

(54) $$q_{4}=q_{9}+q_{10}$$

(55) $$q_{5}+q_{10}=q_{11}+q_{12}$$

(56) $$q_{6}+q_{12}=q_{13}$$

(57) $$q_{7}+q_{8}+q_{19}=q_{20}$$

(58) $$q_{3}+q_{9}=q_{14}+q_{15}$$

(59) $$q_{11}+q_{15}=q_{16}+q_{17}$$

(60) $$q_{13}+q_{17}=q_{18}+q_{19}$$

where *T*_O_, *T*_14_–*T*_20_, and *T*_24_ are the steady-state temperatures of the lenses, support structures, and environment, respectively, and *q*_9_–*q*_20_ are the heat flux rates between nodes.

When *T*_24_ is −38.5 °C and *T*_O_ is 21 °C, the heat leakage rate of the optical system can be calculated using Equations (34)–(60), as illustrated in Table 3. Despite implementing insulation measures between lenses and the frame structure, the heat dissipated by convection and thermal radiation cannot be disregarded. As the optical window directly interfaces with the external environment, it dissipates a significant amount of heat.

## 4. Thermal Optimization Design

### 4.1. Arrangement of Heating Zones

According to the heat leakage analysis results of the optical system, heating zones are arranged at the leakage positions corresponding to the lenses and optical window components. This approach not only maximizes the temperature uniformity of the optical system but also prevents unnecessary heating power consumption.

**Figure 8:**
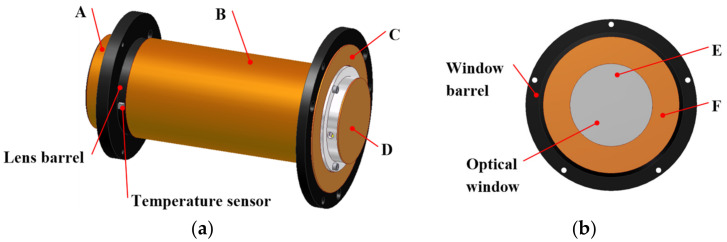
Heating zones of the optical system: (**a**) lens; (**b**) optical window.

As illustrated in Figure 8, four heating zones are established on the lens component and designated as A, B, C, and D, which are located at nodes 12, 8, 13, and 21, respectively. The window component is divided into two heating zones, designated as E and F, which are situated on the outer surface of the window and window barrel, respectively. The lens and window barrel are heated using a silicone heating film, with temperature sensors installed in each heating zone to provide real-time temperature measurements. The optical window is heated by a combination of an indium tin oxide (ITO) conductive film and copper electrode pairs, ensuring that the current flows uniformly through the outer surface of the window. This arrangement prevents the occurrence of local high temperatures, thus mitigating the risk of glass rupture and maintaining the integrity of the optical window.

### 4.2. Thermal Control Strategy

**Figure 9:**
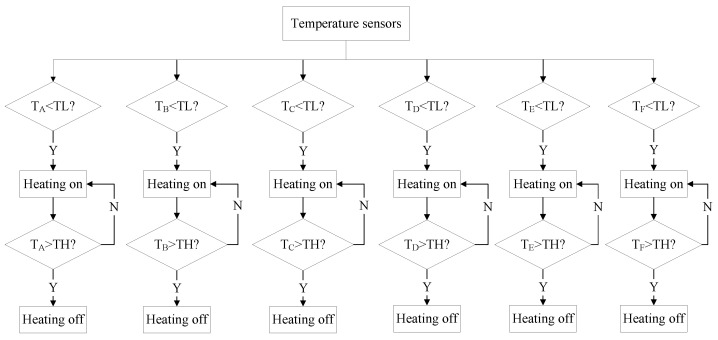
Thermal control strategy.

The bang-bang control algorithm was utilized to independently regulate each heating zone, as depicted in Figure 9. According to the temperature sensor inputs, the heating circuits of each zone are activated or deactivated. Specifically, when the temperature of a certain control zone falls below the set lower point (TL), heating is initiated in that zone. Once the temperature reaches the set upper point (TH), heating is stopped. In this way, the optical system temperature is maintained within the desired range of TL to TH.

The thermal balance equation for the heating zone is

(61) $$q_{g}-q_{\mathrm{out}}=q_{\mathrm{st}}$$

where *q*_g_ is the heating power, *q*_out_ is the heat leakage rate, and *q*_st_ is the internal energy change rate of the unit.

When *q*_g_ exceeds *q*_out_, the internal energy of the unit will increase, leading to a rise in temperature. To meet the temperature requirements, TL and TH are set to 19 °C and 21 °C, respectively. Consequently, the heating power can be adjusted to match the heat leakage rate of the optical system at a temperature of 21 °C and an ambient temperature of −38.5 °C, as specified in Table 2.

## 5. Thermal Control Experiment

**Figure 10:**
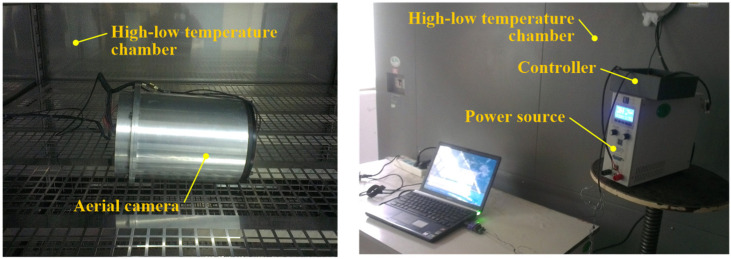
Experimental setup for the thermal control.

**Figure 11:**
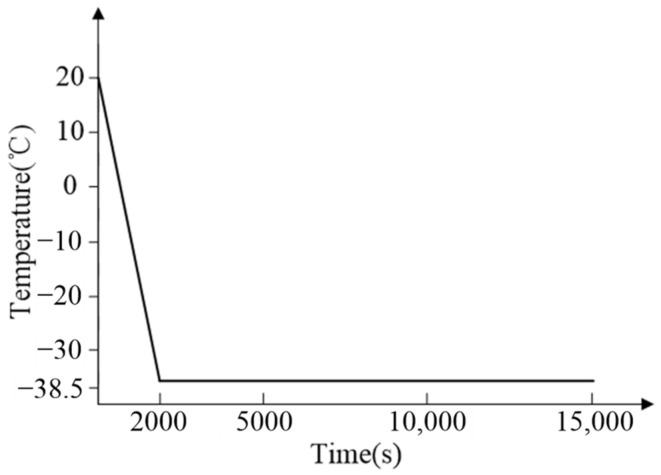
Temperature variations inside the high–low temperature chamber.

To validate the effectiveness of the thermal control design based on our heat leakage analysis, a thermal control experiment is conducted. The aerial camera is situated within a high–low temperature chamber, as illustrated in Figure 10. To simulate the environmental temperature variations experienced during flight conditions, the temperature inside the high–low temperature chamber is set as depicted in Figure 11.

**Figure 12:**
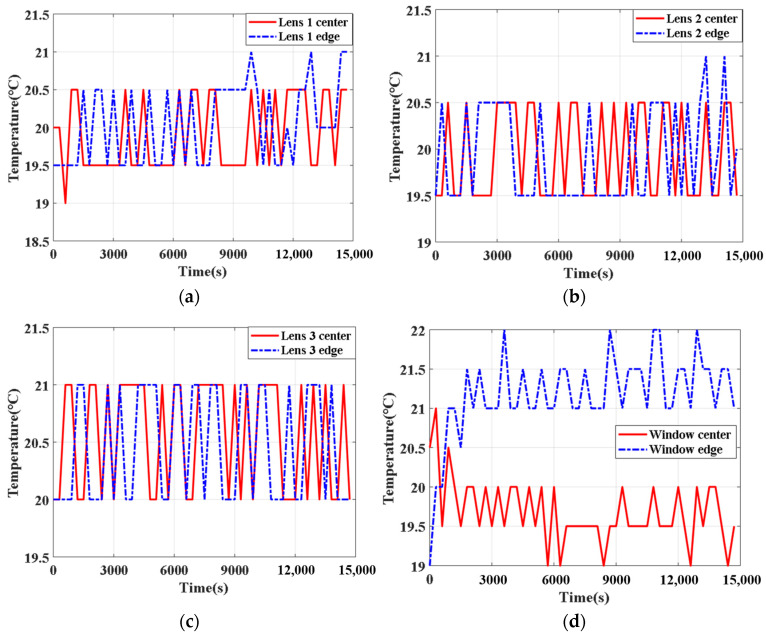
Temperature changes with time of the optical system in the thermal control experiment: (**a**) lens 1; (**b**) lens 2; (**c**) lens 3; (**d**) optical window.

The thermal control system is activated once the temperature inside the high–low temperature chamber begins to vary. Figure 12 presents the temperatures of the optical system changing with time, as measured by the temperature sensors. As can be observed from the figure, during the entire flight duration of the aerial camera, from takeoff to the completion of a shooting task, the temperatures of both lenses and the optical window are maintained within the range of 19 °C to 22 °C, ensuring that the temperature gradients remain below 3 °C. Due to the high-contact thermal resistance between the electrode pairs and the window surface achieved through conductive adhesive, the heat generation at the electrode pairs is relatively high, and the position of the electrode pairs is close to the window edge, resulting in a slightly higher temperature at the window edge than at the center.

## 6. Conclusions

This paper details a thermal optimization design for the optical system of an aerial camera through the development of a sophisticated lumped parameter thermal network (LPTN) model. Optimization is achieved by considering the unique internal structure of the camera, with a focus on enhancing precision by optimizing both convection and radiation thermal resistances. By utilizing the LPTN framework, an intricate heat leakage network is constructed, enabling a thorough analysis of the various heat leakage pathways and rates within the camera system. These insights into the thermal performance of the camera are instrumental in guiding the refinement of heating zones and active thermal control strategies.

It is noteworthy that the thermal control system operates efficiently, with a total power consumption of 26 W, which meets the low power limit of the camera. This efficiency is further demonstrated through experimental results, which show the consistent maintenance of temperatures within the optical system between 19 °C and 22 °C throughout the entire flight. The temperature gradients remain below 3 °C, ensuring optimal performance and stability.

The proposed LPTN model has proven to be highly efficient in acquiring thermal characteristics, significantly simplifying the thermal optimization design process for aerial camera systems. In comparison to previous research, the implementation of the LPTN model proposed in this paper for thermal management has significantly reduced the power consumption of thermal control from several hundred watts to 26 W while also enhancing the temperature control’s accuracy to within 3 °C. This model offers a valuable tool for engineers and designers to quickly and accurately assess thermal performance and make informed decisions regarding thermal management.

## Figures and Tables

**Table 1:** Effects of different temperature levels and temperature gradients on MTF.

Type	Value (°C)	MTF	MTF Requirement
Temperature level	−20	0.506	>0.504
	0	0.513	
	20	0.52	
Radial temperature gradient	3	0.495	>0.480
	5	0.483	
	6	0.475	
Axial temperature gradient	5	0.508	>0.480
	7	0.476	
	10	0.467	

**Table 2:** Materials and physical properties of the thermal network model.

Node Number	Location	Material	Thermal Conductivity/W·m^−1^·°C^−1^	Surface Emissivity	Thermal Capacity/J·°C^−1^
1~3	Lens 1	NBK7	1.114	0.9	4.5/13.5/15.8
4	Lens barrel	LY12	160	0.8	51.1
5~7	Lens 2	NBK7	1.114	0.9	2.3/4.5/6.8
8	Lens barrel	LY12	160	0.8	755.3
9~11	Lens 3	NBK7	1.114	0.9	4.5/15.8/27
12~13	Lens barrel	LY12	160	0.8	121/177.4
14~16	Inner frame	LY12	160	0.07	1216/150.5/930.1
17~19	Outer frame	LY12	160	0.07	414/572.5/1064.4
20	Rear cover	LY12	160	0.07	602.1
21	Lens insulation cover	LY12	160	0.07	48.4
22	Optical window	NBK7	1.114	0.6	144
23	Window barrel	LY12	160	0.8	389.8

**Table 3:** Heat leakage rate of the optical system.

Location	Lenses				Optical Window	
Node	8	12	13	21	22	23
Heat leakage rate (W)	*q*_5_ = 2.8	*q*_4_ = 0.4	*q*_6_ + *q*_7_ = 2.2	*q*_8_ = 1	*q*_1_ = 2	*q*_2_ + *q*_3_ = 17.9
Total heat leakage rate (W)	26.3					

## Data Availability

The data are available from the corresponding author on reasonable request.

## Appendix A

The calculated results of the thermal resistances R_1_–R_43_ from the thermal network equations are shown in Table A1.

**Table A1:** The thermal resistances of the thermal network equations.

*R*_1_	16.78	*R*_2_	11.7	*R*_3_	5.12
*R*_4_	22.52	*R*_5_	17.34	*R*_6_	8.92
*R*_7_	13.5	*R*_8_	8	*R*_9_	2.79
*R*_10_	0.27	*R*_11_	0.37	*R*_12_	0.54
*R*_13_	${\left[0.02{\left|\frac{T_{21}-T_{20}}{T_{21}+T_{20}}\right|}^{0.28}+1.12\times 10^{-11}(T_{21}^{2}+T_{20}^{2})(T_{21}+T_{20})\right]}^{-1}$				
*R*_14_	0.15	*R*_15_	0.11	*R*_16_	51.4
*R*_17_	${\left[0.24{\left|\frac{T_{8}-T_{16}}{T_{8}+T_{16}}\right|}^{0.25}+2\times 10^{-10}(T_{8}^{2}+T_{16}^{2})(T_{8}+T_{16})\right]}^{-1}$				
*R*_18_	51.3	*R*_19_	0.22	*R*_20_	0.22
*R*_21_	51.3	*R*_22_	${\left[0.34{\left|\frac{T_{16}-T_{19}}{T_{16}+T_{19}}\right|}^{0.25}+1.4\times 10^{-10}(T_{16}^{2}+T_{19}^{2})(T_{16}+T_{19})\right]}^{-1}$		
*R*_23_	51	*R*_24_	0.19	*R*_25_	0.23
*R*_26_	${\left[0.07{\left|\frac{T_{13}-T_{20}}{T_{13}+T_{20}}\right|}^{0.28}+3.32\times 10^{-11}(T_{13}^{2}+T_{21}^{2})(T_{13}+T_{21})\right]}^{-1}$				
*R*_27_	${\left[0.02{\left|\frac{\bar{T_{1}}-T_{22}}{\bar{T_{1}}+T_{22}}\right|}^{0.28}+1.73\times 10^{-11}(T_{12}^{2}+T_{22}^{2})(T_{12}+T_{22})\right]}^{-1}$				
*R*_28_	${\left[0.01{\left|\frac{\bar{T_{1}}-T_{22}}{\bar{T_{1}}+T_{22}}\right|}^{0.28}+1.62\times 10^{-11}(T_{4}^{2}+T_{22}^{2})(T_{4}+T_{22})\right]}^{-1}$				
*R*_29_	${\left[0.0093{\left|\frac{\bar{T_{1}}-T_{22}}{\bar{T_{1}}+T_{22}}\right|}^{0.28}+1.37\times 10^{-11}(T_{3}^{2}+T_{22}^{2})(T_{3}+T_{22})\right]}^{-1}$				
*R*_30_	${\left[0.0057{\left|\frac{\bar{T_{1}}-T_{22}}{\bar{T_{1}}+T_{22}}\right|}^{0.28}+8.78\times 10^{-12}(T_{2}^{2}+T_{22}^{2})(T_{2}+T_{22})\right]}^{-1}$				
*R*_31_	${\left[0.0018{\left|\frac{\bar{T_{1}}-T_{22}}{\bar{T_{1}}+T_{22}}\right|}^{0.28}+2.8\times 10^{-12}(T_{1}^{2}+T_{22}^{2})(T_{1}+T_{22})\right]}^{-1}$				
*R*_32_	${\left[0.025+1.14\times 10^{-10}(T_{22}^{2}+T_{24}^{2})(T_{22}+T_{24})\right]}^{-1}$			*R*_33_	11.14
*R*_34_	${\left[0.11+9.62\times 10^{-10}(T_{23}^{2}+T_{24}^{2})(T_{23}+T_{24})\right]}^{-1}$				
*R*_35_	5.12	*R*_36_	${\left[0.038+3.02\times 10^{-11}(T_{17}^{2}+T_{24}^{2})(T_{17}+T_{24})\right]}^{-1}$		
*R*_37_	${\left[0.41+3.24\times 10^{-10}(T_{19}^{2}+T_{24}^{2})(T_{19}+T_{24})\right]}^{-1}$				
*R*_38_	${\left[0.13+1.07\times 10^{-10}(T_{18}^{2}+T_{24}^{2})(T_{18}+T_{24})\right]}^{-1}$				
*R*_39_	${\left[0.23+1.82\times 10^{-10}(T_{20}^{2}+T_{24}^{2})(T_{20}+T_{24})\right]}^{-1}$				
*R*_40_	0.27	*R*_41_	${\left[0.01{\left|\frac{\bar{T_{9}}-T_{21}}{\bar{T_{9}}+T_{21}}\right|}^{0.28}+2.6\times 10^{-12}(T_{11}^{2}+T_{21}^{2})(T_{11}+T_{21})\right]}^{-1}$		
*R*_42_	${\left[0.0058{\left|\frac{\bar{T_{9}}-T_{21}}{\bar{T_{9}}+T_{21}}\right|}^{0.28}+1.8\times 10^{-12}(T_{10}^{2}+T_{21}^{2})(T_{10}+T_{21})\right]}^{-1}$				
*R*_43_	${\left[0.0017{\left|\frac{\bar{T_{9}}-T_{21}}{\bar{T_{9}}+T_{21}}\right|}^{0.28}+5.4\times 10^{-13}(T_{9}^{2}+T_{21}^{2})(T_{9}+T_{21})\right]}^{-1}$				
